# *Core-shell* molecularly imprinted polymer for selective recognition and detection of S-metolachlor in aqueous samples

**DOI:** 10.1038/s41598-026-44780-2

**Published:** 2026-04-01

**Authors:** Dominika Rapacz-Kinas, Katarzyna Smolińska-Kempisty, Joanna Wolska

**Affiliations:** Department of Process Engineering and Technology of Polymer and Carbon Materials, Wroclaw University of Science and Technology, Wybrzeże Wyspiańskiego 27, Wrocław, 50-370 Poland

**Keywords:** *Green* molecularly imprinted polymers, pH-responsible polymers, S-metolachlor, Sorption process, *Core-shell* polymers, Solid phase extraction, Chemistry, Environmental sciences, Materials science

## Abstract

In the present study, an eco-friendly approach for preparing molecularly imprinted polymers (MIPs) applying on poly(vinyl chloride) (PVC) core is reported. The synthesis of pH sensitive MIPs was accomplished utilizing S-metolachlor as a template molecule, acrylic acid and acrylamide as a functional monomers, N,N′- methylenebis(acrylamide) as a cross-linking agent, and water as a porous agent. The morphology and shape of the particles were examined using a scanning electron microscope. The sorbents were subjected to detailed analysis using FT-IR spectroscopy, dynamic light scattering and thermogravimetric analysis techniques. The synthesized CS-MIPs exhibited a high degree of selectivity towards the target molecule S-metolachlor, sorbing it more than twice as effectively as atrazine and 2,4-D. The obtained CS-MIPs were successfully employed to the detection of S-metolachlor in both model and real samples. A methodology for the regeneration of sorbents has been developed, allowing for the reuse of the same material several times.

## Introduction

 The utilization of coupled analytical techniques has been identified as a highly effective method for the detection and separation of traces of various analytes, including pharmaceuticals, heavy metals, pesticides, and industrial or household chemicals, in aquatic ecosystems^1–3^. The combination of liquid chromatography and mass spectrometry is widely applied in the qualitative and quantitative analysis of a variety of micropollutants present in complex samples^3^. Nevertheless, this method demands the expertise of skilled technicians, the employment of costly instrumentation, and time-consuming sample pretreatment procedures^4–6^.

As a cost-effective approach, the use of molecularly imprinted polymers (MIPs) as a selective analyte extraction material is proposed. During the polymerization process of MIPs, specific binding sites are created on the polymer frameworks. Then, the template molecules are removed, and molecular cavities are generated that can recognize and capture only those analytes that fit them specially. These binding sites are complementary to the target analyte in their morphology, size and molecular interactions^7^. The selection of appropriate monomers, termed functional monomers, for polymerization is of significance in increasing the selectivity of the molecular imprinting process. These monomers possess recognition sites, such as heteroatoms, functional groups, and π-π conjugated systems, which interact with the binding sites of template molecules through covalent bonds, and non-covalent bonds like hydrogen bonds, electrostatic attractions, π-π stacking, and van der Walls and hydrophobic interactions^8,9^. MIPs are applied in a wide range of fields, including medicine, biochemistry, biology, chromatography, solid-phase extraction, separation techniques, imaging methods, immunoassays, drug delivery systems, and optical and electrochemical sensors, due to their specific properties^9–12^.

Despite many advantages associated with MIPs, conventional methods of synthesizing these compounds are regarded as non-eco-friendly. In the traditional synthesis process, a considerable volume of organic solvents is employed, in addition to the utilization of toxic chemicals. This has the potential to pose a health risk to operators, generate substantial chemical waste, and have a detrimental effect on the environment^3^. Accordingly, a novel eco-friendly approach for MIPs synthesis is gaining popularity, including the use of renewable, innocuous reagents, the control of polymerization without energy waste, and the utilization of aqueous media as porogen and solvent. Furthermore, the design of self-cleaning MIPs, enhanced degradability of MIPs at environmental conditions after use, implementation of polymerization at mild conditions, and optimization via computational modelling are assumptions of the greenification concept^13^.

Traditionally, MIPs are synthesized by bulk polymerization. However, the irregular size and small surface area of the obtained polymers, as well as the heterogeneous binding sites, render this method less attractive^14^. As an alternative strategy, the MIPs layer can be grown as a shell on a nanoparticle core, creating *core-shell* particles. This type of structure undoubtedly exhibits several notable advantages, including uniform morphology, homogeneous binding site distribution, and a comparatively large surface area^15^. The combination of materials that form *core-shell* particles results in the demonstration of remarkable properties, which could not be attained through the utilisation of the constituent materials in their separate forms^16^.

Herbicides are the most commonly used pesticides in agriculture for the management of weeds. Nevertheless, the synthesis of these compounds results in significant environmental and water contamination^17^. The presence of trace amounts of pesticides in water, ranging from undetectable to a few micrograms per liter, has been termed micropollutants^18^. S-metolachlor ((*S*)-2-Chloro-*N*-(2-ethyl-6-methyl-phenyl)-*N*-(1-methoxypropan-2-yl)acetamide) is one of the most widely used herbicide in the cultivation of maize^19^. The absorption of S-metolachlor by soil particles is facilitated by a range of interactions, including Van der Waals forces, hydrophobic partitioning, charge-transfer complexes, ligand exchange, and covalent bonding. These interactions may occur individually or in combination, depending on the specific conditions of the environment^20^. The herbicidal activity of metolachlor is caused primarily by S-isomers, which are now widely employed due to their enhanced herbicidal activity in comparison to their racemate forms^21^.

In the present study, pH-responsible *core-shell* polymers with S-metolachlor imprints in the shell have been prepared according to the strategies of *greenification*. More specifically, the pre-polymerization mixture consists of acrylic acid, sensitive to pH, and acrylamide as a functional monomers, N,N′- methylenebis(acrylamide) as a cross-linking agent, and water as a porous agent. The resultant mixture was then applied as a layer on the poly(vinyl chloride) core. In this paper, we describe the design and preparation of *core-shell* particles and their applications as functional sorbents of S-metolachlor from an aqueous samples.

## Materials and methods

### Reagents and chemicals

The following chemicals were utilized in the synthesis of the *core-shell* polymers: acrylamide (AA), obtained from Thermo Scientific Chemicals; acrylic acid (AAc), received from Acros Organics; ammonium persulfate (APS), N,N′-methylenebis(acrylamide) (BIS), and N, N,N’,N’-tetramethylethylenediamine (TEMED) purchased from Sigma-Aldrich. S-metolachlor (SMCh) was received from NOVAGRA. Atrazine was supplied by ABCR GmbH & Co. 2,4-dichlorophenoxyacetic acid (2,4-D) was received from Corteva. Poly(vinyl chloride) (Ongrovil^®^ S-5167) was purchased from BorsodChem, with medium molecular weight, and with a K value (an indicator of molecular weight) of 66–68. Ethyl alcohol (EtOH) and the HPLC grade acetonitrile was supplied by POCH S.A. Ultrapure water was obtained with a Milli-Q System (Merck Millipore).

### Dehydrochlorination of PVC

The process of dehydrochlorination of PVC was conducted in accordance with the methodology delineated by Wolska et al.^22^ To summarize the procedure, 67.5 g of PVC was dispersed in a 500 mL 10% KOH solution (4:1 w/w isopropyl alcohol: water). The mixture was then subjected to heating at its boiling point under the reflux condenser for a period of 24 h. During this process, the color of the reaction mixture underwent a transition from a colorless to a dark brown hue. Subsequently, the degraded PVC was filtered and washed with a large volume of water, followed by a final wash with ethanol. The obtained product was extracted with ethanol for 24 h. Following this extraction process, DHPVC was subjected to drying at a temperature of 60 °C in a dryer.

### Amination of DHPVC

The amination of PVC was undertaken in accordance with the methodology established by Wolska et al.^22^ A quantity of 25 g of DHPVC was added to 200 mL of amine in a 500 mL flask. The flask containing the mixture was then subjected to heating at its boiling point for a period of 10 min and subsequently stored in a reaction mixture for 24 h at room temperature. Then, the resin was filtered, washed with distilled water to remove ethylenediamine (EDA), and dried in a 60 °C a dryer.

### Preparation of core-shell polymers

The preparation of *core-shell* molecularly imprinted polymers was achieved through the following procedure. Approximately 5 g of poly(vinyl chloride) grains, which had undergone the amination process, were contacted with 14 mL of an aqueous solution of the prepolymerization mixture. This mixture consists of the two functional monomers: acrylic acid and acrylamide (5.76–19.98 mmol and 5.84–19.98 mmol, respectively), as well as a cross-linking agent N, N′-methylenebis (acrylamide) (4.93-5.00 mmol), and S-metolachlor (0.27–0.40 mmol). The prepolymerization mixture was subsequently sonicated for a period of 10–15 min in order to dissolve BIS. Thereafter, 15.3 mL of water was added to the reactor and the mixture was left to incubate for a further 10–15 min. In the next step of the process, the prepolymerization mixture was purged with nitrogen for 5–10 min. Following this, the initiator ammonium persulfate (0.20–0.30 mmol) and tetramethylethylenediamine (0.15–0.23 mmol), was added. The reaction was conducted for 24 h at ambient temperature. The mixture was subsequently washed onto Buchner funnel and extracted on a Soxhlet apparatus for a period of 24 h. Following this, the polymers were dried at room temperature. The non-imprinted *core-shell* polymers (CS-NIPs) was synthesized in an identical way to CS-MIPs, with the exception that S-metolachlor molecules were not added during the process. The compositions of individual polymers are presented in Table 1.

**Table 1:** The composition of the polymers.

CS-MIPs marking	AAc (mmol)	AA (mmol)	BIS (mmol)	SMCh (mmol)	APS (mmol)	TEMED (mmol)
1	5.76	6.96	4.93	0.40	0.30	0.23
2	6.87	5.84	4.93	0.40	0.20	0.15
3	19.98	19.98	5.00	0.27	0.20	0.15

### Analytical and characterization methods

#### *High performance liquid chromatography*

High performance liquid chromatography (HPLC) system (Shimadzu high performance liquid chromatograph model SCL-40) equipped with an autosampler (model SIL-40), a photodiode array (PDA) detector and a chromatographic column (3 μm ARION^®^ Biphenyl, 150 × 4.6 mm) was used. Isocratic elution was carried out at a temperature of 30 °C, with a mixture of water and acetonitrile (50:50 v/v) employed as the mobile phase. The maintenance of a flow rate of 1 mL × min^− 1^ was achieved, with an injection volume of 30 µL and a detection wavelength of 254 nm. The mobile phase was degassed by sonication for 10 min.

#### *Scanning electron microscopy*

The characterization of polymer morphologies and structures was performed by scanning electron microscopy (SEM). The samples were mounted on aluminum stubs using double-sided carbon tape. Following this, the specimens were gold-coated for 400 s by utilizing an Edwards Scancoat Six Sputter Coater. A scanning electron microscope (Zeiss Evo LS15) operating at 16 kV was used for imaging.

#### *Fourier-transform infrared spectroscopy*

The characterization of the chemical structures of the polymers was performed by Fourier-transform infrared spectroscopy (FT-IR). The acquisition of spectral data was conducted on a Jasco FT-IR-4700 spectrophotometer, employing a total of 64 scans with a resolution of 4 cm^− 1^ within the 400–4000 cm^− 1^ spectral range.

#### Thermogravimetric analysis

The thermal stability characteristics of CS-MIPs and CS-NIPs were investigated by thermogravimetric analysis (TGA). This analysis was conducted using the TGA 8000 apparatus from Perkin Elmer. The heating of the polymers was performed within an nitrogen atmosphere, with a constant flow rate of 10 mL × min^− 1^, ranging from 30 to 1000 °C. The rate of heating was maintained at 10 °C × min^− 1^.

#### Dynamic light scattering

Determination of size distribution of PVC, CS-MIPs, and CS-NIPs was achieved through implementation of a dynamic light scattering (DLS) technique. The analysis was carried out using the LS 13 320 XR Laser Diffraction Particle Size Analyzer. The measurements were obtained by dispersing particles in water, with this process repeated three times. SPAN number was calculated as

$$SPAN=\frac{{d}_{90}-{d}_{10}}{{d}_{50}}$$

where ${d}_{90}$, ${d}_{50},{d}_{10}$ are the diameters for 90, 50, and 10% of the particle population^23^.

#### Porosity of sorbents

The specific surface area (${S}_{BET}$) and pore volume (${V}_{N2}$) were determined through the analysis of nitrogen adsorption at the liquid nitrogen temperature, utilizing Autosorb IQ gas sorption analyzer (Quantachrome, Boynton Beach, FL, USA). Before analysis, degassing of the samples was conducted at a temperature of 50 °C for duration of 10 h. The nitrogen dosage was set at 3 cm^3^ × g^− 1^. The free space of the analytical tube was measured before performing the analysis.

### Adsorption experiments

#### Batch mode sorption process

For all batch mode sorption experiments, a quantity of sorbents (CS-MIPs and CS-NIPs) with a mass of approximately 0.05–0.2 g was weighed into the flask. Thereafter, 30 mL f a solution of S-metolachlor at a concentration of 30 mg × L^− 1^ was added and the contents of the flask were stirred for a period of 24 h. The concentration of the solution after sorption was then determined by HPLC. Sorption capacity ($q$) – defined as the amount of S-metolachlor adsorbed at the equilibrium – was calculated using following equation:

1 $$q= \frac{(C_0-C_eq)\times V}{m}$$

where ${C}_{0}$ and ${C}_{eq}$ (mg × L^− 1^) are the concentrations of the herbicide at the initial solution and at the equilibrium, respectively, m (g) is the mass of the dry sorbents, V (L) is a volume of the solution^24^.

#### Dynamic mode sorption on the SPE columns

For all dynamic mode sorption experiments, conducted on the SPE columns, approximately 0.05 g of sorbents (CS-MIPs and CS-NIPs) was weighed and placed into the SPE columns. Subsequently, the sorbents were washed with water to dampen the surface. Thereafter, 2 mL of S-metolachlor solution was added and incubated with the sorbents for varying periods of time. The concentration of the solution after sorption was then determined by HPLC, and the sorption was calculated according to Eq. 1.

#### Competitive study

An investigation was conducted into the binding selectivity of the polymers from multicomponent tap water solution (pH~7.9), consisting of the herbicides: atrazine, glyphosate and S-metolachlor (concentration: 15 mmol × L^− 1^) in dynamic mode. The introduction of approximately 0.050 ± 0.001 g of sorbent (CS-MIPs or CS-NIPs) into a 1 mL SPE column and activation with water was performed. Thereafter, the column was incubated for 10 min with 2 mL of a S-metolachlor solution with a concentration of 30 mg × L^− 1^. The solution concentration was determined by HPLC.

The calculation of the distribution coefficient ($K$) was undertaken in order to characterize the selective properties, according to the following equation:

2 $${K}_{i\left(j\right)}=\frac{{q}_{i\left(j\right)}\times\rho}{{C}_{i\left(j\right)eq}}$$

where ${q}_{i\left(j\right)}$ (µg × g^− 1^) is the sorption capacity ($i$ – S-metolachlor, $j$ – other plant protection products), $\rho$(g × L^− 1^ ) is the density of the solution, and ${C}_{i\left(j\right)eq}$ (µg × L^− 1^) is the equilibrium concentration ($i$ – S-metolachlor, $j$ – other plant protection products^25^.

Moreover, the calculation of the imprinting factor ($IF$) was conducted using Eq. 3:

3 $$IF=\frac{{K}_{i}^{MIP}}{{K}_{i}^{NIP}}$$

where *K*_*i(j)*_ is the distribution coefficient ($i$ – S-metolachlor, $j$ – other plant protection products) of an analyte on an imprinted polymer (${K}^{MIP}$) and a non-imprinted polymer (${K}^{NIP}$)^25^.

The specific selectivity factor ($S$) was calculated according to the following equation (Eq. 4):

4 $$S=\frac{{IF}_{i}}{{IF}_{j}}$$

where ${IF}_{i}$ and ${IF}_{j}$ are imprinting factor of the S-metolachlor and other plant protection products, respectively^25^.

#### Sorption from real samples

The sorption study was conducted on both the model solution and the sample, which were prepared to simulate the properties of a real sample, in dynamic mode. Approximately 0.050 ± 0.001 g of sorbent (CS-MIPs or CS-NIPs) was introduced into a 1 mL SPE column and activated with water. Thereafter, the column was incubated for 10 min with 2 mL of a S-metolachlor solution with a concentration of 30 mg × L^− 1^. The concentration of the solution was determined by HPLC, and the sorption was calculated according to Eq. 1.

#### Bed regeneration

The estimation of the regeneration factor (${R}_{f}$) of the deposit was conducted through the implementation of the following equation (Eq. 5):

5 $${R}_{f}=\frac{{m}_{e}}{{m}_{a}}\times100\%$$

where ${m}_{e}$ (mg) is mass of eluted S-metolachlor, and ${m}_{a}$ (mg) is mass of absorbed S-metolachlor.

## Results and discussion

### Characterization of adsorbents

#### FT-IR analysis

**Fig. 1:**
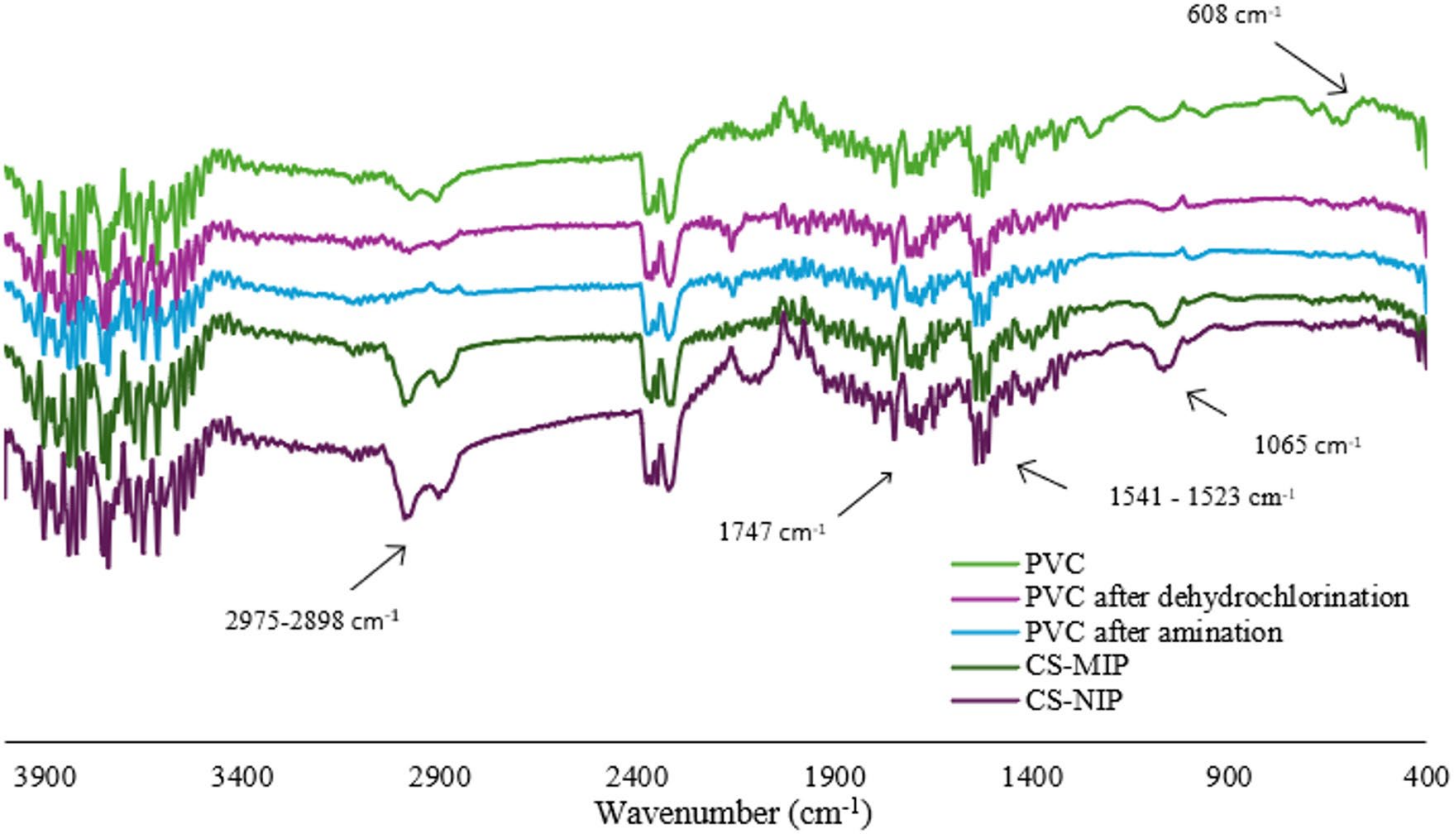
The FT-IR spectra of the core (PVC), and *core-shell* structures (CS-MIPs and CS-NIPs).

FT-IR spectroscopy was performed to further confirm the grafting of molecularly imprinted polymer and non-imprinted polymer layer on the poly(vinyl chloride) core. The results are illustrated in Fig. 1. Notably, bands in the range of 2975 cm^− 1^ and 2898 cm^− 1^ were observed in the spectra of PVC, CS-MIPs, and CS-NIPs, which could indicate aliphatic C–H vibrations. Furthermore, both CS-MIPs and CS-NIPs showed a peak at 1747 cm^− 1^ which is presumably attributable to C=O vibrations. The presence of the amine group is likely to be responsible for the peaks within the range of 1541 cm^− 1^ and 1523 cm^− 1^. The observed band at 1065 cm^− 1^ could be indicative of the presence of C–N bonding. In the PVC spectra, the C–Cl band was identified at 608 cm^− 1^. After the amination process of PVC, spectra exhibited bands at 1020 cm^− 1^ which are probably related to the presence of the amine group.

#### SEM analysis

**Fig. 2:**
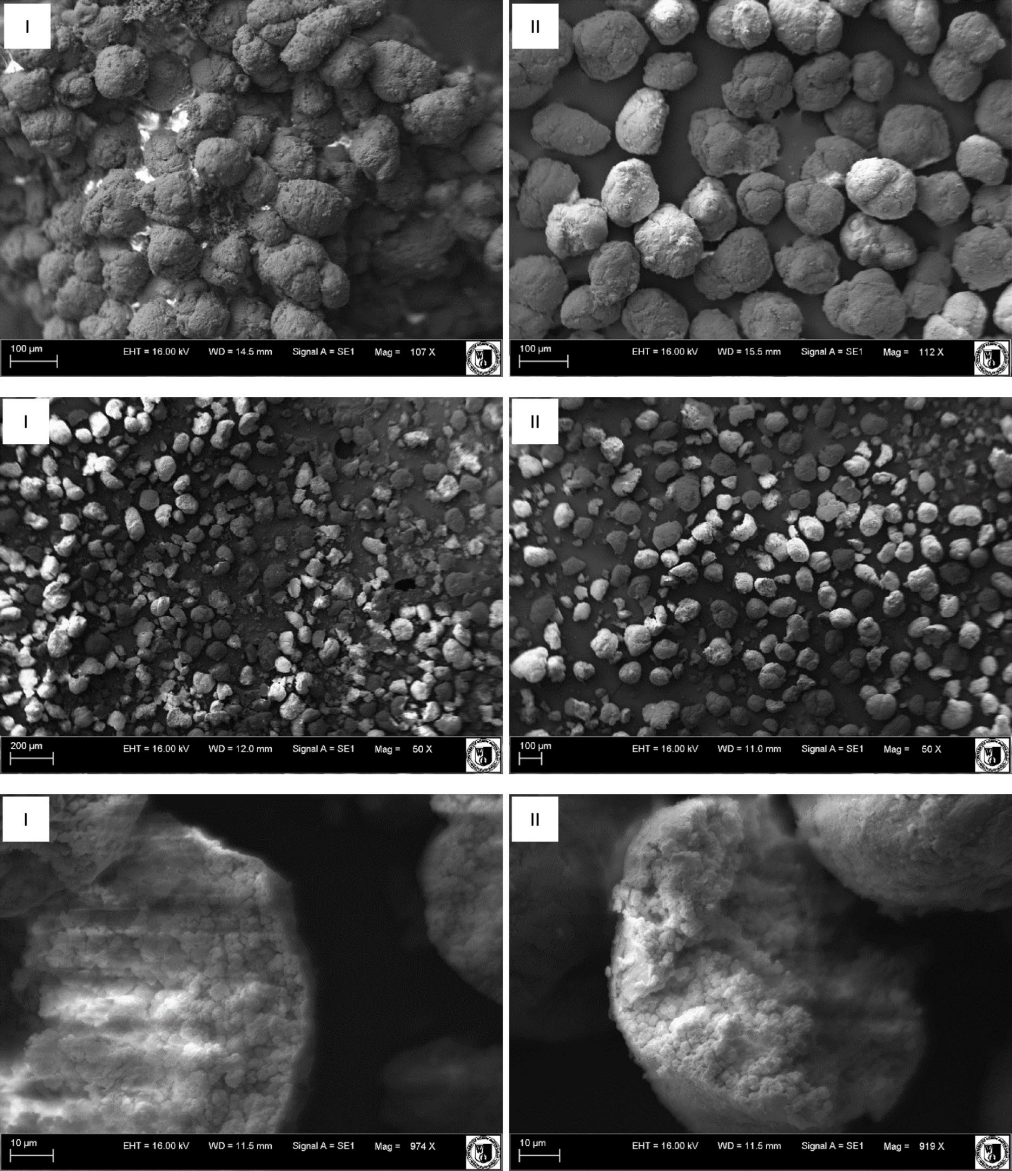
The scanning electron microscope images of: CS-MIPs (I); CS-NIPs (II).

The structure and morphology of the obtained materials are important parts for the exploration of their properties. The SEM images of CS-MIPs (I) and CS-NIPs (II) are shown in Fig. 2. It can be seen that CS-MIPs and CS-NIPs particles exhibit comparable sizes, however, their surfaces are characterized by a rough and irregular texture.

#### TGA analysis

**Fig. 3:**
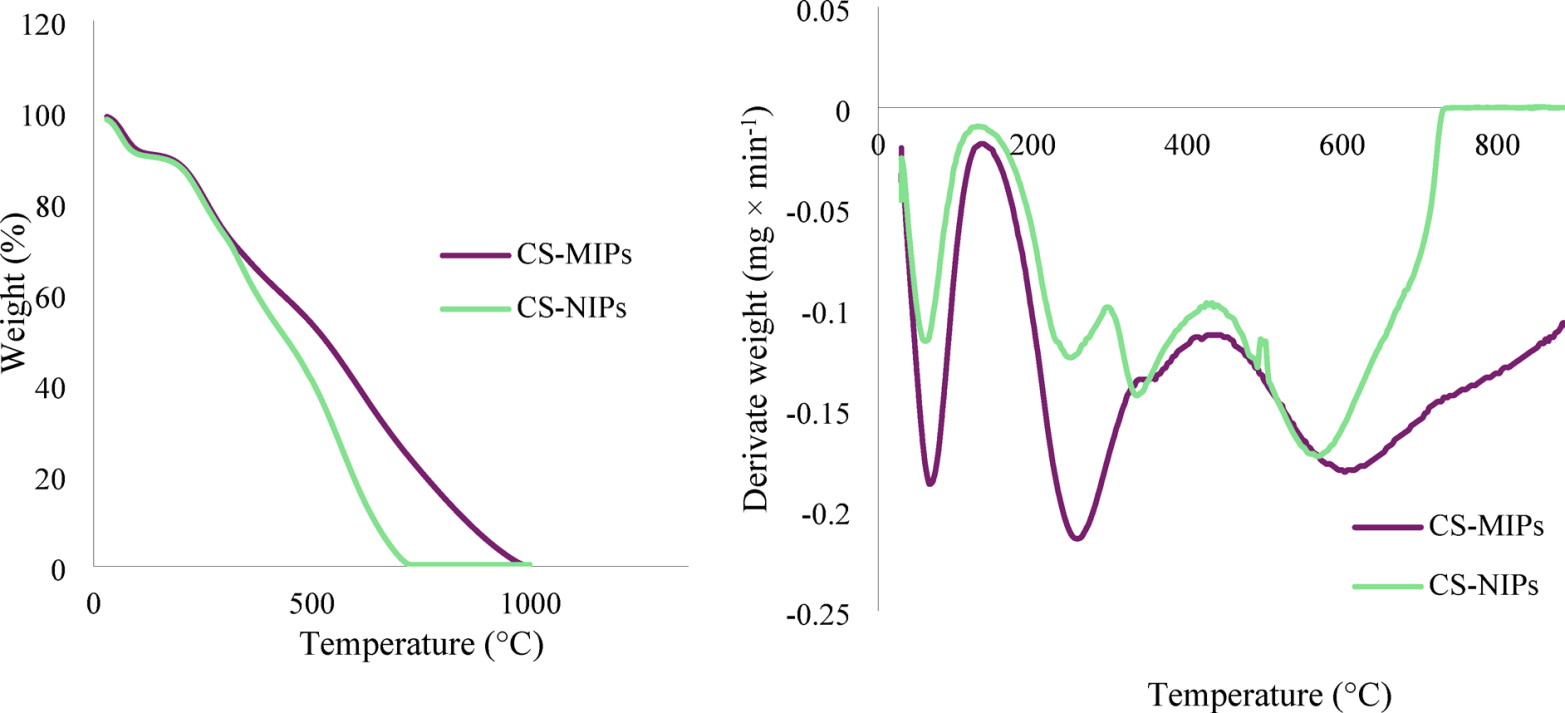
The results of TGA analysis for CS-MIPs and CS-NIPs.

The thermal stability of the synthesized polymers was investigated by thermogravimetric analysis. Initially, up to a temperature of approximately 315 °C, the graphs for CS-MIPs and CS-NIPs appear almost identical. It is evident that both polymers undergo a loss of water in the initial phase, up to approximately 100 °C, with the most significant loss of mass occurring at approximately 230 °C. For CS-NIPs, the loss of mass in the temperature range from approximately 700 °C to 1000 °C indicates the destruction of the polymers, while for CS-MIPs, complete mass loss was achieved at approximately 1000 °C (Fig. 3).

#### Dynamic light scattering

The average diameter of the sorbent particles and the SPAN number were measured using the dynamic light scattering method The mean diameter of the PVC particles following the amination process was found to be 162.1 μm, with a SPAN value of 0.74. For CS-MIPs particles, the average diameter was measured to be 150.2 μm, and for CS-NIPs it was 117.7 μm. The SPAN value for CS-MIPs was 0.50, and for CS-NIPs it was 0.81. The PVC particles were composed of two different batches, each exhibiting varied average diameters. It can be assumed that the batch containing smaller grains was utilised for the synthesis of CS-NIPs, which would account for the observed decrease in their average diameter and faster decomposition of the sorbent, as evident in the graph.

#### Porosity study

The porosity of the materials was studied (including PVC after amination process, CS-MIPs and CS-NIPs) in order to ascertain their specific surface area and pore volume. The results of the study are presented in Table 2. Following the application of a layer of molecularly imprinted polymers to the core, there was a decrease in the surface area of CS-NIPs, as the pores present on the surface of PVC were probably occluded. However, in the case of CS-MIPs, an increase in the specific surface area was observed, thereby possibly confirming the presence of selective binding cavities. Due to the small surface area, it was not possible to determine the average pore size.

**Table 2:** The porosity study data.

PVC	${\boldsymbol{S}}_{\boldsymbol{B}\boldsymbol{E}\boldsymbol{T}}$ (m^3^ × g^− 1^)	${\boldsymbol{V}}_{\boldsymbol{N}2}$ (cm^3^ × g^− 1^)
	5.8	0.018
CS-MIPs	7.4	0.019
CS-NIPs	3.4	0.017

### Optimizing the condition of the synthesis process

**Fig. 4:**
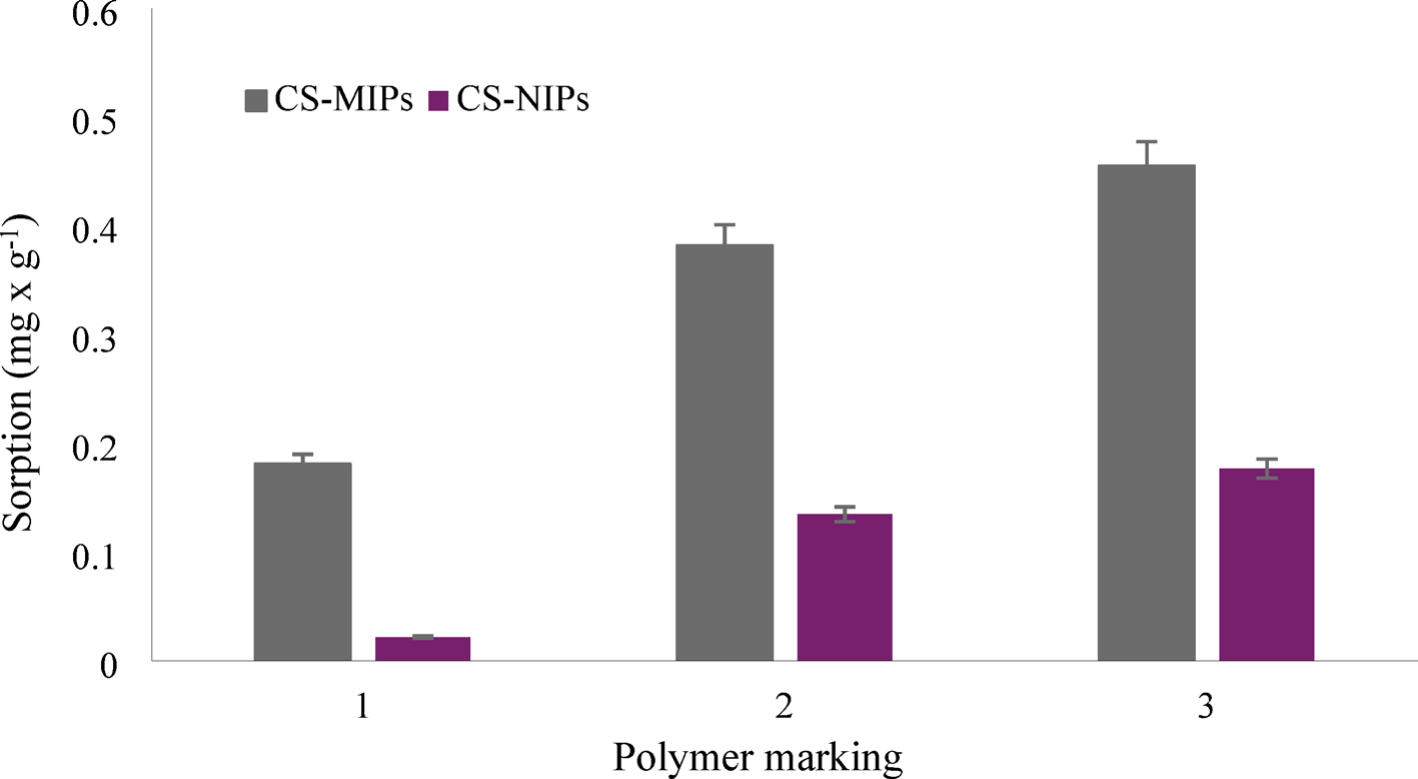
The results of sorption process.

The synthesis process was carried out in compliance with the procedure described in paragraph 2.4 and the experience gained from our previous studies^26^. All of the *core-shell* polymers obtained exhibit the ability to adsorb S-metolachlor. However, for sorbent marked as 1 (see Fig. 4), the sorption ratio for CS-MIPs and CS-NIPs is 8.23, while for sorbent 2 it is 2.83, and for sorbent 3 it is 1.74. Given that CS-MIPs sorb S-metolachlor about 8 times better than CS-NIPs, sorbent marked as 1 was selected for the further, more detailed studies. In a subsequent section of this work, the selected *core-shell* composition with molecular imprint will be designated as CS-MIPs, while the molecule without molecular imprint will be designated as CS-NIPs.

One of the parameters investigated was the number of the polymer layers applied on the poly(vinyl chloride) core. The results are presented in Table 3. The sorption of S-metolachlor by CS-MIPs after the application of one layer of polymer to the PVC core is greater than after the application of two layers of polymer. Moreover, the difference between sorption by CS-MIPs and CS-NIPs is more significant when only one layer is applied. In addition, the lower consumption of reactants and the reduced synthesis time were taken into consideration, which together indicated that the optimal application would be one layer of polymer on the core particle.

**Table 3:** The results of the optimization of the number of applied polymer layers.

1 layer of applied polymer	Sorption of CS-MIPs (mg × g^− 1^)	Sorption of CS-NIPs (mg × g^− 1^)
	0.364 ± 0.028	0.201 ± 0.034
2 layers of applied polymer	0.287 ± 0.033	0.183 ± 0.035

Another parameter that was optimized was the pH of the solution from which sorption is carried out (see Table 4). In all pH ranges examined, CS-MIPs exhibited approximately twofold enhanced sorption capacity for S-metolachlor in comparison to CS-NIPs, thereby confirming the efficacy of sorption processes for this herbicide within the pH range of 2.5 to 9.0. However, given that the material is intended for use in the detection of S-metolachlor from real samples, and IF value was the most favorable, further studies were conducted at a pH of approximately 7.9.

**Table 4:** Sorption values for CS-MIPs and CS-NIPs from solutions at different pH.

pH	Sorption (mg × g^− 1^)		IF
	CS-MIPs	CS-NIPs	
2.5	0.35 ± 0.04	0.16 ± 0.01	2.2
4.5	0.41 ± 0.10	0.17 ± 0.11	2.4
7.9	0.18 ± 0.01	0.02 ± 0.02	9.0
9	0.32 ± 0.08	0.15 ± 0.05	2.1

### Optimization of sorption time

**Fig. 5:**
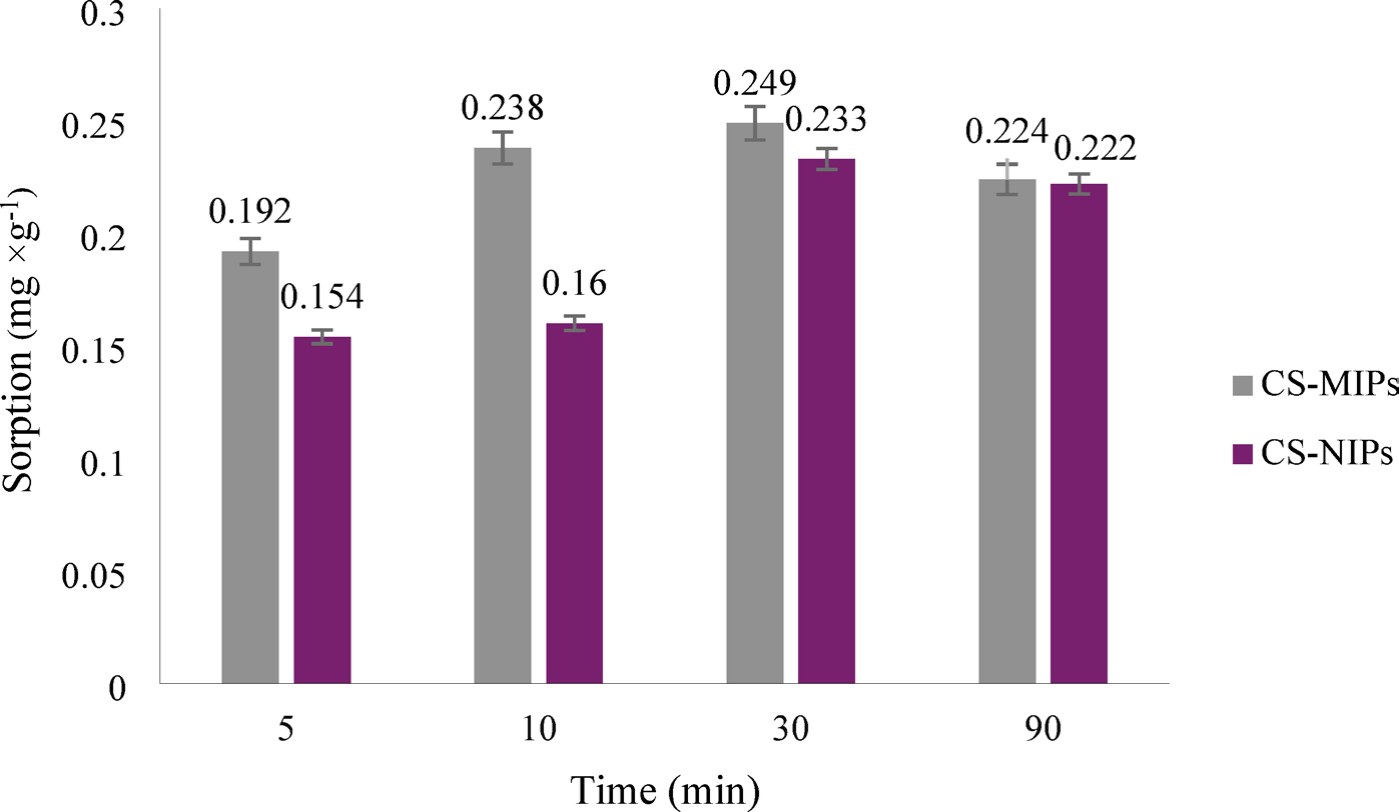
The results of the optimization of the incubation time on the SPE columns.

A crucial parameter that necessitated optimization during dynamic sorption on SPE columns was the incubation time of the bed with the S-metolachlor solution. The results of the research are presented in Fig. 5. Following analysis of the data presented below, it was determined that 10 min of contact between the sorbents and the S-metolachlor solution would be the optimal time, as the difference in sorption values for CS-MIPs and CS-NIPs after this time is the greatest. Moreover, the reduced experimental time permits almost immediate analysis of the sample.

### Sorption and desorption cycles

The regeneration properties of the bed were also examined to ascertain the number of times the material could be reused. The pure ethanol was employed as an eluent. The results obtained can be found in Table 5. As illustrated by the data presented above, the CS-MIPs can be utilized up to three times while maintaining its sorption properties. This has the advantage of reducing the cost of production, analysis and obtaining a favorable environmental impact.

**Table 5:** The results of the sorption–desorption process.

Sorption I (mg × g^− 1^)	CS-MIPs	CS-NIPs
	0.35 ± 0.07	0.22 ± 0.09
${R}_{f}$ (%)	96 ± 4	95 ± 2
sorption II (mg × g^− 1^)	0.28 ± 0.02	0.16 ± 0.02
${R}_{f}$ (%)	95 ± 5	97 ± 3
sorption III (mg × g^− 1^)	0.39 ± 0.15	0.31 ± 0.11
${R}_{f}$ (%)	98 ± 2	99 ± 1

### Selectivity study

In addition to the difference in sorption values between CS-MIPs and CS-NIPs, it is critically important that the synthesized material exhibits selective sorption of S-metolachlor. In order to achieve this objective, a solution comprising various plant protection agents was formulated: atrazine, the commercially available Mustang containing 2,4-dichlorophenoxyacetic acid, and S-metolachlor. The results obtained are collated in Table 6. Despite the application of a thin layer of molecularly printed polymer to the PVC core, the material exhibits high selectivity, with the ability to absorb S-metolachlor over two times more effectively than atrazine and 2,4-D.

**Table 6:** The results of the selectivity study.

Parameter	S-metolachlor	Atrazine	2,4-D
*IF*	3.034	1.389	1.452
*S*	–	2.18	2.09

### Sorption from real samples

In order to verify that the resulting material sorbs S-metolachlor not only from the model solution, but also from real samples, a simulated real solution was prepared from tap water. The sorption process was conducted in dynamic mode, and the results obtained are presented in Table 7. The data presented demonstrates the efficacy of the material in absorbing S-metolachlor from both types of solution. In addition, following the principles of greenification, it is possible to reduce the mass of the sorbent used by a factor of 4, while maintaining the material’s high affinity for S-metolachlor.

**Table 7:** The sorption results of S-metolachlor from both model and real solutions.

CS-MIPs^1^	Sorption from model solution (mg × g^− 1^)	Sorption from real samples (mg × g^− 1^)
	0.24 ± 0.01	0.28 ± 0.02
CS-NIPs^1^	0.12 ± 0.01	0.08 ± 0.02
CS-MIPs^2^	0.18 ± 0.01	0.18 ± 0.05
CS-NIPs^2^	0.02 ± 0.02	0.07 ± 0.03

^1^The data for the weighting of 0.2 g

^2^The data for the weighting of 0.05 g

### Method validation

The analytical parameters of the HPLC method, including the limit of detection (LOD), limit of quantification (LOQ), the regression coefficient (R^2^), and the linearity range have been ascertained. The establishment of a linear calibration plot was undertaken, the regression coefficient of which was equal 0.9999. This objective was achieved through the correlation of the peak area with the concentration of S-metolachlor, within the range of 0.141–215.016 mg × L^− 1^. The LOD was determined to be 0.113 mg × L^− 1^, indicating the minimum concentration at which the S-metolachlor can be detected but not reliably quantified. The LOQ was estimated to be 0.142 mg × L^− 1^, representing the lowest concentration of the analyte that could be accurately quantified. The calibration curve was described by the formula y = 3703.87x.

## Conclusions

The *core-shell* structure combined with molecularly imprinted polymers exhibit a specific recognition ability for S-metolachlor. Based on the results, it could be concluded that CS-MIPs could selectively isolate S-metolachlor from both model and simulated real solutions. Furthermore, the strong binding affinity of CS-MIPs for S-metolachlor was well established even after three times adsorption–desorption cycles. The reusability of the sorbents and synthesis method is indicative of the *greenification* of MIPs. The optimum conditions of S-metolachlor sorption on CS-MIPs on SPE columns were achieved at an incubation time of 10 min. In addition, the potential applications of presented CS-MIPs in the rapid separation of S-metolachlor, as well as in the purification and enrichment of the water samples, are promising. The sorbents were characterized using a range of techniques, including FT-IR, SEM, TGA and DLS.

## Data Availability

Data will be made available upon request.
